# Changes in the proteomic profiles of mouse brain after infection with cyst-forming *Toxoplasma gondii*

**DOI:** 10.1186/1756-3305-6-96

**Published:** 2013-04-12

**Authors:** Dong-Hui Zhou, Fu-Rong Zhao, Si-Yang Huang, Min-Jun Xu, Hui-Qun Song, Chunlei Su, Xing-Quan Zhu

**Affiliations:** 1 State Key Laboratory of Veterinary Etiological Biology, Key Laboratory of Veterinary Parasitology of Gansu Province, Lanzhou Veterinary Research Institute, Chinese Academy of Agricultural Sciences, Lanzhou, Gansu Province 730046, PR China; 2 Department of Microbiology, The University of Tennessee, Knoxville, TN 37996, USA; 3 College of Animal Science and Veterinary Medicine, Heilongjiang Bayi Agricultural University, Daqing, Heilongjiang Province 163319, PR China

**Keywords:** *Toxoplasma gondii*, Cyst, Brain, Proteome, Two-dimensional gel electrophoresis (2-DE), Mass spectrometry (MS)

## Abstract

**Background:**

*Toxoplasma gondii* is an opportunistic pathogenic protozoan parasite, which infects approximately one third of the human population worldwide, causing opportunistic zoonotic toxoplasmosis. The predilection of *T. gondii* for the central nervous system (CNS) causes behavioral disorders and fatal necrotizing encephalitis and thus constitutes a major threat especially to AIDS patients.

**Methods:**

In the present study, we explored the proteomic profiles of brain tissues of the specific pathogen-free (SPF) Kunming mice at 7 d, 14 d and 21 d after infection with cysts of the *Toxoplasma gondii* Prugniaud (PRU) strain (Genotype II), by two-dimensional gel electrophoresis (2-DE) combined with MALDI-TOF/TOF tandem mass spectrometry (MS/MS).

**Results:**

A total of 60 differentially expressed protein spots were selected. Fifty-six spots were successfully identified, which corresponded to 45 proteins of the mouse. Functional analysis using a Gene Ontology database showed that these proteins were mainly involved in metabolism, cell structure, signal transduction and immune responses, and will be beneficial for the understanding of molecular mechanisms of *T. gondii* pathogenesis.

**Conclusions:**

This study identified some mouse brain proteins involved in the response with cyst-forming *T. gondii* PRU strain. These results provided an insight into the responsive relationship between *T. gondii* and the host brain tissues, which will shed light on our understanding of the mechanisms of pathogenesis in toxoplasmic encephalitis, and facilitate the discovery of new methods of diagnosis, prevention, control and treatment of toxoplasmic encephalopathy.

## Background

The obligate intracellular parasite *Toxoplasma gondii* is an important water- and food-borne protozoan and can infect humans as well as almost all warm-blooded animals including mammals and birds, and the infection usually persists throughout the life of the hosts [1-5]. *T. gondii* infects approximately 30% of the human population worldwide and 8% of population in China [6]. Humans can be infected by ingesting tissues cysts in uncooked meat or by ingesting food and water contaminated with oocysts from infected cat feces [1,2,6]. There are three infectious stages of *T. gondii*: tachyzoites (the rapidly multiplying form), bradyzoites (latent form found in tissue cysts) and sporozoites (in oocysts) [7]. Bradyzoites develop in cysts within host cells in a number of tissues, and they are more common in neural and muscular tissues.

*T. gondii* has long been known as an important opportunistic pathogen of immuno-compromised patients. Toxoplasmosis ranks high on the list of diseases that lead to death of the AIDS patients. Encephalitis caused by *T. gondii* is the most predominant manifestation of toxoplasmosis in immunosuppressed patients and is now recognized with great frequency in patients treated with immunosuppressive agents [8-11].

In the past decade, proteomic approaches have been extensively employed to study the interaction between pathogens and their hosts. The most frequently used technique in quantitative proteomics is two-dimensional electrophoresis (2-DE). In quantitative 2-DE, the appropriate experimental design plays an important role in the detection of significant and reliable protein expression differences [12]. Despite that there are some limitations of the technology, such as offering a limited dynamic range of separated proteins, it has been used to investigate host cell proteome changes after infection with *T. gondii* tachyzoites [13,14], but little is currently known of the proteomic changes at differential time points in host brain tissues after infection with *T. gondii* cysts. In the present study, we applied 2-DE combined with mass spectrometry to study proteomic changes in mouse brain tissues infected with *T. gondii* cysts. The objective was to examine the proteomic modulation of host brain by cyst-forming *T. gondii in vivo*.

## Methods

### Ethics statement

The present study was approved by the Animal Ethics Committee of Lanzhou Veterinary Research Institute, Chinese Academy of Agricultural Sciences (Approval No. LVRIAEC2010-008). The mice were handled in accordance with good animal practices required by the Animal Ethics Procedures and Guidelines of the People’s Republic of China.

### Sample collection

Sixty Specific-Pathogen-Free (SPF)-grade female Kunming mice (including 30 mice for *T. gondii* infection and 30 mice as non-infected control), aged 6 to 8 weeks old, were purchased from Sun Yat-Sen University Laboratory Animal Center. All mice were handled in accordance with good animal practice according to the Animal Ethics Procedures and Guidelines of the People’s Republic of China. All mice were maintained under standard conventional conditions, with food and water *ad libitum*.

*T. gondii* (PRU) strain (Genotype II) was kindly provided by Prof Hai-Zhu Zhang (Department of Parasitology, Xinxiang Medical College, Henan, China) and were preserved in our laboratory. Tissue cysts of the PRU strain were obtained from the brains of Kunming mice infected with cysts according to Yan *et al*. [15]. Cysts were counted under an optical microscope. After counting, each of the 30 mice in the treatment group was inoculated intra-gastrically with 10 cysts of the PRU strain, while 30 mice in the non-infection control group were inoculated intra-gastrically with sterile physiological saline. After inoculation, the mice were observed daily for clinical symptoms. Based on a previous study [16] and our pilot study, at 7 d, 14 d and 21 d post infection, six mice from both treatment group and the control group were euthanized and the whole brain of each mouse was rapidly removed separately, washed to remove the blood, and immediately stored at −80°C until proteomic analysis, or directly processed for protein extraction. The mice that were successfully infected and produced *T. gondii* cysts were determined by observing tissue cysts in the brain under an optical microscope (for 14 d and 21 d).

### Protein extraction

Proteins were prepared according to the previously published protocol [13]. In brief, mouse brain tissues of six mice from each group, respectively, were lysed in lysis buffer containing 7 M urea, 2% CHAPS {3-[(3-cholamidopropyl)-dimethylammonio]-1-propanesulfonate}, 2 M thiourea (Amersham), 20 mM Tris–HCl (pH 8.5, Amresco) and phenylmethylsulfonyl fluoride solution (Amresco). Then the sample was sonicated on ice (80 W, 12 s duration, 10 times, with 2 min intervals) and centrifuged at 12,000 × *g* for 20 min at 4°C. The supernatant was transferred to a new centrifuge tube with four times the volume of acetone added. The mixture was then precipitated overnight at −20°C and centrifuged on the next day with the same parameters. The precipitate was harvested and stored at −80°C until use. The samples were prepared in triplicates.

### Isoelectric focusing electrophoresis

2-DE procedure was performed essentially according to a protocol published previously [13]. Briefly, before the isoelectric focusing electrophoresis, the precipitate was dissolved in rehydration buffer containing 7 M urea, 2% CHAPS, 2 M thiourea, followed by centrifugation at 12,000 × *g* for 20 min at 4°C. The protein concentration was determined by the Bradford method using a 2D Quant kit (Amresco) according to the manufacturer’s instructions. Using an equal mixture of the brain tissue protein of each mouse in the control group and treatment group respectively, proteins were initially separated using an Ettan IPGphor 3 Isoelectric Focusing system (GE Healthcare). Brain tissue proteins were focused to their isoelectric points on a 24 cm (pH 4–7) Immobiline DryStrip (GE Healthcare) with the following parameters: 300 V for 20 min, 700 V for 30 min, 1,500 V for 1.5 h, 9,000 V for 3 h and 9,000 V for 4 h. After the isoelectric focusing, the IPG strip was equilibrated for 15 min in equilibration buffer containing 2% sodium dodecyl sulfate (SDS), 50 mM Tris–HCl (pH 8.8), 6 M urea, 30% (vol/vol) glycerol, 0.002% bromophenol blue and 100 mM dithiothreitol (freshly added before use, Amresco), and followed by a second wash for 15 min with equilibration buffer containing 250 mM iodoacetamide (freshly added before use, Amersham). The IPG strip was then embedded in a precast gel and sealed into place using agarose sealing solution.

### SDS-PAGE

After equilibration, the immobilized pH gradient strips were loaded onto 12.5% (w/v) homogeneous acrylamide gels (1 mm × 24 cm × 19 cm) and sealed with 1% (w/v) agarose. Proteins were separated by running the gels at 2 W/gel for 45 min and then at 18 W/gel at 10°C until the bromochlorophenol blue reached the end of gels. Finally, the gels were fixed in fixing solution (ethanol: glacial acetic acid: deionized water = 4:1:5) for 2 h, stained with Coomassie Brilliant Blue G-250 overnight and rinsed with deionized water.

### Analysis of gels

Images of gels were obtained at 150 dpi (dots/in) using a scanner (Powerlook1100, UMAX) and analyzed using ImageMaster™ 2D Platinum 5.0 software (GE Healthcare). Spots were detected by 2DElite Automatic Spot Detection Program, which calculated spot volumes relative to the background and normalization. The volume percentage of each spot was determined by comparison of the spot volume to the total volume presented in the 2-DE gel. To select differentially expressed protein spots, quantitative analysis was performed using the Student’s *t*-test by the volume percentage of spots between brain tissues of the infected and uninfected groups on the triplicate gels. Two spots were considered significantly different if *P* < 0.05 and with 1.5 fold differences in volume. Spots meeting these criteria were then selected and subjected to in-gel tryptic digestion.

### Protein enzymolysis

The differentially expressed protein spots were manually excised from the Coomassie Brilliant Blue-stained gels and put into a 96-well microplate. The gel pieces were washed twice with MilliQ water, destained with 50% methanol at 37°C for 30 min, and dehydrated in 100 μl of acetonitrile (ACN) at room temperature for 20 min. Next, the samples were swollen in 50 μl of 100 mM NH_4_HCO_3_, dehydrated for the second time and incubated in 1 μg/50 μl trypsin (Promega) at 4°C for 30 min. Then the samples were added with coverage solution (10% ACN, 50 mM NH_4_HCO_3_, MilliQ water) and incubated at 37°C for 16 h. After suction of the coverage solution, the peptide mixtures were extracted using 2.5% trifluoroacetic (TFA)/90% ACN at room temperature for 30 min and vacuum dried.

### Identification of protein spots by MS

After vacuum drying, material was dissolved in 1.5 μl solution containing MilliQ water, 50% ACN and 0.1% TFA. Then, 0.8 μl of the mixture was loaded onto a target plate with 0.5 μl HCCA (5 mg/ml a-Cyano-4-hydroxycinnamic acid) matrix, dried at room temperature and analyzed using ABI 4800 matrix-assisted laser desorption ionization-time of flight/time of flight (MALDI-TOF/TOF) Proteomics Analyzer mass spectrometer (Applied Biosystems, USA). The UV laser was operated at a 200 Hz repetition rate with a wavelength of 355 nm and an accelerated voltage of 20 kV.

### Database searching

The experimental MS data were matched to a corresponding virtual peptide mass database derived from GPS Explorer™ v 3.6, the Mascot and the International Protein Index (IPI) mouse protein database. Protein identification was carried out by peptide mass fingerprint (PMF) using the Mascot software (http://www.matrixscience.com). The search parameters used in PMF were as follows, database: IPI mouse (56871 sequences); species: mouse; enzyme: trypsin; fixed modifications: carbamidomethylation; variable modifications: oxidation (M). The function, gene name, and Gene Ontology category of each protein were determined using the Mascot v 2.1 software protein database search engine and the IPI mouse protein database.

### Quantitative real-time PCR verification

Total RNA was extracted from the mouse brain tissues infected with *T. gondii* cysts using the Trizol reagent (Invitrogen). One microgram of total RNA was used to synthesize the first-strand cDNA, which was diluted 20 fold; 5 μl of the diluted cDNA was used as a template for real time PCR. SYBR Green-monitored real time PCR was performed on an ABI PRISM® 7500 Sequence Detection System (Applied Biosystems). The primer sequences used for real time PCR are listed in Table 1. Glyceraldehyde-3-phosphate dehydrogenase (GAPDH) was used as a housekeeping gene for the tests.

**Table 1:** Primer sequences and amplicon lengths of quantitative real-time PCR products of target genes

**Target genes**	**Primers (5′ to 3′)**	**Amplicon length (bp)**
Calreticulin	FP: CACCAAGAAGGTTCATGTCA	190
	RP: CAGAAAGTCCCAATCATCCT	
Rho GDP-dissociation inhibitor 1	FP: AGTCTTGTGACCCCGGAAGT	129
	RP: TCTGCCATGCTTACCTCTAGC	
Endoplasmin	FP: ACCAGACACCAAGGCGTATG	150
	RP: TCTCCCTCATCCTGCTCTGA	
Stomatin-like protein 2	FP: GTCAGCGCATTCTCCAAACT	180
	RP: CGTGTCTGTAGCCTGGACAT	
GAPDH	FP: CGGCCTCCAAGGAGTAAGAAA	141
	RP: GCCCCTCCTGTTATTATGG	

FP: Forward primer; RP: Reverse primer.

## Results

### Comparative proteomic analysis by 2-DE

Global protein components of mouse brain tissues were separated with 2-DE in a 24 cm, pH 4–7 IPG strip. The 2-DE gels were processed by silver staining or Coomassie brilliant blue G-250 staining, then scanned using a UMAX scanner. Consistency of the method was confirmed by analyzing in gels in triplicate. The data were analyzed using ImageMaster™ 2D Platinum 5.0 software. The results showed that at least 2500 protein spots were detected in each gel. Spots with significant increase (or decrease) in their relative abundance were considered the differentially expressed proteins if *P <* 0.05 and two spots have 1.5 fold differences in volume. After bioinformatics analysis, 60 differentially expressed proteins were selected and identified using MALDI-TOF MS.

### Identification of differentially expressed proteins

Proteins with MW ranging from 17 to 170 kDa and pI between 4 and 7 were separated well. Differentially expressed proteins were picked from gels and identified using MALDI-TOF MS. The data are summarized in Table 2. Sixty significantly and consistently up- or down-regulated protein spots with 1.5 fold changes of volume intensity in the triplicate gels were digested by trypsin and analyzed by MALDI-TOF MS.

**Table 2:** **Differentially expressed proteins between *T. gondii*-infected and non-infected mouse brain tissues**

**Spot No.**	**Accession No.**	**Protein name**	**Score**	**Expect**	**Queries matched**	**Sequence Coverage**	**Nominal mass**	**Calculated pI value**	**Matched peptides**	**Fold change (Infected/non-infected)**^**A**^	**Functional categories**^**B**^
**Day 7, down-regulated proteins**											
1	IPI00230394	Lamin-B1	288	9.00E-25	27	36	66973	5.11	ASAPATPLSPTR;LQEKEELR;SLETENSALQLQVTEREEVR;ALYETELADARR;LREYEAALNSK;VDLENRCQSLTEDLEFRKNMYEEEINETRR;LVEVDSGR;LAQALHEMREQHDAQVRLYKEELEQTYHAK;LALDMEISAYR;LKNTSEQDQPMGGWEMIR;NQNSWGTGEDVK;NSQGEEVAQR;TTIPEEEEEEEEEPIGVAVEEERFHQQGAPR	−2.91	Structural molecule activity
2	IPI00944143	Serpin B6 isoform a	218	9.00E-18	9	25	45201	5.99	SRPGCCAGPRGYR;NVFLSPMSISSALAMVFMGAK;TGTQYLLR;FYEAELEELDFQGATEESR;FIEWTR;LGMTDAFGGR;AFVEVNEEGTEAAAATAGMMTVR	−1.68	Enzyme regulator activity and metabolic process
3	IPI00110721	Isoform 1 of Glyoxalase domain-containing protein 4	508	9.00E-47	12	41	33581	5.28	FQTVHFFRDVLGMQVLRHEEFEEGCK;LGNDFMGITLASSQAVSNAR;VAEGIFETEAPGGYKFYLQDR;IYEQDEEKQR;LELQGIQGAVDHAAAFGR;ELPDLEDLMKR;LLDDAMEADKSDEWFATR	−1.51	Cell part and organelle
4	IPI00113660	Proteasome activator complex subunit 3	471	4.50E-43	9	42	29602	5.69	ITSEAEDLVANFFPK;RLDECEEAFQGTK;SNQQLVDIIEKVKPEIR;IEDGNNFGVSIQEETVAELRTVESEAASYLDQISRYYITR;YPHVEDYRRTVTEIDEKEYISLR	−1.70	Enzyme regulator activity
5	IPI00130344	Chloride intracellular channel protein 1	202	3.60E-16	6	39	27338	5.09	IGNCPFSQR;YPKLAALNPESNTSGLDIFAK;VLDNYLTSPLPEEVDETSAEDEGISQR;GFTIPEAFR;YLSNAYAREEFASTCPDDEEIELAYEQVAR	−1.89	Transporter activity
6	IPI00121851	Prefoldin subunit 3	133	2.90E-09	4	15	22592	6	FLLADNLYCK;NLDSLEEDLDFLR;VYNWDVKR	−1.51	Metabolic process
7	IPI00315794	Cytochrome b5	212	3.60E-17	6	54	16365	4.79	VEGSEPSVTYYR;NSAEETWMVIHGRVYDITRFLSEHPGGEEVLLEQAGADATESFEDVGHSPDAR;QYYIGDVHPSDLKPK	−1.70	Binding and enzyme activator activity
8	IPI00124501	Galectin-related protein A	257	1.10E-21	8	57	19172	5.12	LDDGHLNNSLGSPVQADVYFPRLIVPFCGHIK;AVFTDR;NSCISGERGEEQSAIPYFPFIPDQPFRVEILCEHPR;VFVDGHQLFDFYHRIQTLSAIDTIK	−1.74	Binding
9	IPI00308984	Eukaryotic translation initiation factor 1A	248	9.00E-21	3	28	16564	5.07	ELVFKEDGQEYAQVIK;LEAMCFDGVKR;VWINTSDIILVGLR	−1.67	Binding
**Day 7, up-regulated proteins**											
10	IPI00131830	Serine protease inhibitor A3K	470	5.70E-43	9	28	47021	5.05	DLQILAEFHEK;ALYQTEAFTADFQQPTEAK;ELISELDER;ISFDPQDTFESEFYLDEKR;HFRDEELSCSVLELK;MQQVEASLQPETLRK;FSIASNYR;AVLDVAETGTEAAAATGVIGGIR	2.55	Enzyme regulator activity
11	IPI00131830	Serine protease inhibitor A3K	511	4.50E-47	12	35	47021	5.05	DLQILAEFHEK;ALYQTEAFTADFQQPTEAK;ELISELDER;ISFDPQDTFESEFYLDEKR;HFRDEELSCSVLELK;MQQVEASLQPETLRK;FSIASNYRLEEDVLPEMGIKEVFTEQADLSGITETK;AVLDVAETGTEAAAATGVIGGIR	3.15	Enzyme regulator activity
12	IPI00131830	Serine protease inhibitor A3K	463	2.90E-42	10	28	47021	5.05	DLQILAEFHEK;ALYQTEAFTADFQQPTEAK;ELISELDER;ISFDPQDTFESEFYLDEKR;HFRDEELSCSVLELK;MQQVEASLQPETLRK;FSIASNYR;AVLDVAETGTEAAAATGVIGGIR	6.30	Enzyme regulator activity
13	IPI00131830	Serine protease inhibitor A3K	564	2.30E-52	14	35	47021	5.05	DLQILAEFHEK;ALYQTEAFTADFQQPTEAK;ELISELDER;ISFDPQDTFESEFYLDEKR;HFRDEELSCSVLELK;MQQVEASLQPETLRK;FSIASNYRLEEDVLPEMGIKEVFTEQADLSGITETKK;AVLDVAETGTEAAAATGVIGGIR	4.02	Enzyme regulator activity
14	IPI00131830	Serine protease inhibitor A3K	596	1.40E-55	12	35	47021	5.05	DLQILAEFHEK;ALYQTEAFTADFQQPTEAK;ELISELDER;ISFDPQDTFESEFYLDEKR;HFRDEELSCSVLELK;MQQVEASLQPETLRK;FSIASNYRLEEDVLPEMGIKEVFTEQADLSGITETK;AVLDVAETGTEAAAATGVIGGIR	5.80	Enzyme regulator activity
15	IPI00310972	GTP-GDP dissociation stimulator 1 isoform b	490	5.70E-45	18	27	61381	5.28	NEFMR;DQEVLLQTGR;EQFASTNIAEELVK;QIEHDKREMIFEVLAPLAENDAIK;LMDLLDRHVEDGNVTVQHAALSALR;SEMPPVQFK;MLIDAQAEAAEQLGK;LVEWCEAKDHAGVMGESNR;DLASAQLVQILHRLLADER;SVAQQASLTEQR	8.32	Cell part and organelle part
**Day 14, down-regulated proteins**											
16	IPI00131871	COP9 signalosome complex subunit 4	449	7.20E-41	18	50	46541	5.57	QLLTDFCTHLPNLPDSTAK;VISFEEQVASIRQHLASIYEKEEDWRNAAQVLVGIPLETGQKQYNVDYKLETYLK;LYLEDDDPVQAEAYINRASLLQNESTNEQLQIHYK;KFIEAAQR;TIVHESERLEALKHALHCTILASAGQQR;MLATLFKDERCQQLAAYGILEK;ATTADGSSILDR;IASQMITEGRMNGFIDQIDGIVHFETR	−2.50	Metabolic process
17	IPI00338039	Tubulin beta-2A chain	591	4.50E-55	22	42	50274	4.78	MREIVHIQAGQCGNQIGAKFWEVISDEHGIDPTGSYHGDSDLQLERINVYYNEAAGNK;AILVDLEPGTMDSVR;GHYTEGAELVDSVLDVVRK;IREEYPDRIMNTFSVMPSPK;FPGQLNADLRKLAVNMVPFPRLHFFMPGFAPLTSRGSQQYRALTVPELTQQMFDSKNMMAACDPR;ISEQFTAMFRR	−1.90	Structural molecule activity
18	IPI00113660	Proteasome activator complex subunit 3	450	5.70E-41	11	43	29602	5.69	ERITSEAEDLVANFFPK;RLDECEEAFQGTK;SNQQLVDIIEKVKPEIR;IEDGNNFGVSIQEETVAELRTVESEAASYLDQISRYYITR;YPHVEDYRRTVTEIDEKEYISLR	−2.04	Enzyme regulator activity
19	IPI00407019	Tetratricopeptide repeat protein 19	348	9.00E-31	12	23	41550	5.87	LSIMKDEPEAAELILHDALRLAYESDNRK;GQLENAEQLFK;QLSQAQR;ALQICQEIQGER;EIYQEALKR;RDEVSVQHIREELAELSR	−1.99	Cell part, organelle and organelle part
20	IPI00133440	Prohibitin	688	9.00E-65	14	42	29859	5.57	AVIFDRFR;DLQNVNITLRILFRPVASQLPRIYTSIGEDYDERVLPSITTEILK;FDAGELITQR;QVSDDLTER;QVAQQEAER;AAELIANSLATAGDGLIELRKLEAAEDIAYQLSR	−4.73	Metabolic process
21	IPI00313962	Ubiquitin carboxyl-terminal hydrolase isozyme L1	340	5.70E-30	8	39	25165	5.14	LGVAGQWR;QIEELKGQEVSPK;QFLSETEKLSPEDR;CFEKNEAIQAAHDSVAQEGQCR;MPFPVNHGASSEDSLLQDAAK;EFTEREQGEVR	−1.68	Cell proliferation
22	IPI00322312	Rho GDP-dissociation inhibitor 1	536	1.40E-49	12	49	23450	5.12	SIQEIQELDKDDESLR;YKEALLGRVAVSADPNVPNVIVTR;EGVEYR;YIQHTYR;IDKTDYMVGSYGPRAEEYEFLTPMEEAPK;FTDDDKTDHLSWEWNLTIK	−4.10	Enzyme regulator activity
23	IPI00845712	Isoform 1 of UPF0424 protein C1orf128 homolog	540	5.70E-50	17	73	24405	5.48	CAAEREEPPEQRGLAYGLYLR;LQCLNESR;GVFKPWEER;FVESDADEELLFNIPFTGNVKLKGVIIMGEDDDSHPSEMR;NIPQMSFDDTEREPEQTFSLNRDITGELEYATK;IFYIGLRGEWTELRRHEVTICNYEASANPADHRVHQVTPQTHFIS;	−2.40	Cell part, organelle and organelle part
24	IPI00221826	Isoform Short of Splicing factor, arginine/serine-rich 3	226	1.40E-18	7	41	14422	10.12	DSCPLDCK;AFGYYGPLRSVWVARNPPGFAFVEFEDPR;NRGPPPSWGR;DDYRR	−1000000	Metabolic process
**Day 14, up-regulated proteins**											
25	IPI00123613	Protein kinase C and casein kinase substrate in neurons protein 1	382	3.60E-34	15	40	50886	5.15	LCNDLMSCVQER;AYAQQLTDWAKR;AWGAMMTEADKVSELHQEVK;AYHLACKEER;TTPQYMEGMEQVFEQCQQFEEKR;HLNLAENSSYMHVYRELEQAIRGADAQEDLR;KAEGATLSNATGAVESTSQAGDR;ALYDYDGQEQDELSFK;LGEEDEQGWCRGRLDSGQLGLYPANYVEAI	4.65	Catalytic activity and binding
26	IPI00110265	Isoform 1 of NAD-dependent deacetylase sirtuin-2	255	1.80E-21	11	35	43856	5.23	NLFTQTLGLGSQK;LLDELTLEGVTRYMQSER;VICLVGAGISTSAGIPDFR;HPEPFFALAK;CYTQNIDTLER;IFSEATPRCEQCQSVVKPDIVFFGENLPSR;APLATPR;ELEDLVRREHANIDAQSGSQAPNPSTTISPGK	1.57	Catalytic activity and binding
27	IPI00649438	Novel protein similar to splicing factor, arginine/serine-rich 3	80	0.00061	3	19	19404	11.41	NPPGFAFVEFEDPRDAADAVR;NRGLPPSWGR	1.65	Metabolic process
**Day 21, down-regulated proteins**											
28	IPI00133522	Protein disulfide-isomerase	408	9.00E-37	17	37	57507	4.79	SNFEEALAAHK;ALAPEYAKR;LKAEGSEIR;VDATEESDLAQQYGVR;YQLDKDGVVLFKKFDEGRNNFEGEITK;HNQLPLVIEFTEQTAPK;ILFIFIDSDHTDNQR;KEECPAVR;YKPESDELTAEKITEFCHR;IKPHLMSQEVPEDWDKQPVKVLVGANFEEVAFDEKK;LGETYKDHENIIIAK;FFPASADR	−1.52	Metabolic process
29	IPI00756786	SH3-domain GRB2-like endophilin B2	258	9.00E-22	11	26	45165	5.44	KLASDAGIFFTR;FGQAEKTELDAHFENLLAR;NWTER;QTEVLLQPNPSAR;ATCEGDTVPDFQETRPR;VAQTEFDRQAEVTRLLLEGISSTHVNHLR;SQTTYYAQCYR	−1.50	Cellular process
30	IPI00111556	Serine/threonine-protein phosphatase 2A catalytic subunit beta isoform	239	7.20E-20	10	34	36123	5.21	SPDTNYLFMGDYVDR;QITQVYGFYDECLRKYGNANVWK;LQEVPHEGPMCDLLWSDPDDRGGWGISPR;AHQLVMEGYNWCHDRNVVTIFSAPNYCYR;YSFLQFDPAPR	−2.10	Metabolic process
31	IPI00798544	Isoform 2 of Enolase-phosphatase E1	384	2.30E-34	11	24	25457	4.92	QLQGHMWK;MKAEFFADVVPAVRRWREAGMKVYIYSSGSVEAQK;GHKVDSESYRK	−3.54	Metabolic process
32	IPI00230145	Ferritin heavy chain	408	9.00E-37	11	65	21224	5.53	QNYHQDAEAAINR;YFLHQSHEEREHAEK;KPDRDDWESGLNAMECALHLEKSVNQSLLELHKLATDKNDPHLCDFIETYYLSEQVK;ELGDHVTNLRKMGAPEAGMAEYLFDKHTLGHGDES	−1.56	Immune system process
**Day 21, up-regulated proteins**											
33	IPI00403810	Tubulin alpha-1C chain	174	2.30E-13	5	16	50562	4.96	AVFVDLEPTVIDEVR;EIIDLVLDR;NLDIERPTYTNLNR;FDGALNVDLTEFQTNLVPYPRIHFPLATYAPVISAEK	1.56	Structural molecule activity
34	IPI00221540	Erlin-2	339	7.20E-30	8	17	38077	5.37	SVQTTLQTDEVK;VTKPNIPEAIRR;VAQVAEITYGQK;KISEIEDAAFLAR;AKADAECYTALK	1.85	Metabolic process
35	IPI00230192	Isoform Alpha-1 of Guanine nucleotide-binding protein G(o) subunit alpha	170	5.70E-13	5	16	40629	5.34	AMDTLGVEYGDKER;LWGDSGIQECFNR;YYLDSLDRIGAGDYQPTEQDILR;LFDVGGQR	1.54	Signal transducer activity
36	IPI00653664	Isoform 1 of Enolase-phosphatase E1	365	1.80E-32	11	27	28696	4.79	EYLQTHWEEEECQQDVSLLR;QLQGHMWK;MKAEFFADVVPAVRRWREAGMKVYIYSSGSVEAQK;VDSESYRK	1.96	Metabolic process
37	IPI00130280	ATP synthase subunit alpha	376	1.40E-33	11	18	59830	9.22	TGTAEMSSILEERILGADTSVDLEETGRVLSIGDGIAR;NVQAEEMVEFSSGLK;TGAIVDVPVGEELLGR;APGIIPRISVREPMQTGIKAVDSLVPIGR;ELI IGDR	2.43	Transporter activity and developmental process
38	IPI00129161	Somatotropin	201	4.50E-16	14	45	24986	5.97	AQHLHQLAADTYKEFERAYIPEGQR;EEAQQRTDMELLR;IFTNSLMFGTSDR;LKDLEEGIQALMQELEDGSPR;QTYDKFDANMR;AETYLR;RFVESSCAF	19.73	Cell proliferation
**Spots 39–46 were down-regulated on both day 14 and day 21**											
39	IPI00119113	V-type proton ATPase subunit B	641	4.50E-60	34	64	56857	5.57	MALRAMRGIVNGAAPELPVPTGGPMAGAR;NYLSQPR;YAEIVHLTLPDGTKR;AVVQVFEGTSGIDAKKTSCEFTGDILRTPVSEDMLGR;GPVVLAEDFLDIMGQPINPQCRIYPEEMIQTGISAIDGMNSIAR;IPIFSAAGLPHNEIAAQICR;SKDVVDYSEENFAIVFAAMGVNMETAR;LALTTAEFLAYQCEKHVLVILTDMSSYAEALR;RGFPGYMYTDLATIYER;QIYPPINVLPSLSRLMKSAIGEGMTR;DHADVSNQLYACYAIGK;AVVGEEALTSDDLLYLEFLQK;NFITQGPYENRTVYETLDIGWQLLR;RIPQSTLSEFYPR	−2.11	Transporter activity
40	IPI00759870	Isoform 4 of Heterogeneous nuclear ribonucleoproteins C1/C2	201	4.50E-16	3	9	32261	4.95	GFAFVQYVNER;MYSYPARVPPPPPIAR	−1.52	Metabolic process
41	IPI00115117	Stomatin-like protein 2	231	4.50E-19	12	32	38475	8.95	NTVILFVPQQEAWVVER;ILEPGLNVLIPVLDR;ASYGVEDPEYAVTQLAQTTMR;YEIKDIHVPPRVKESMQMQVEAER;ATVLESEGTR;APVPGAQNSSQSRRDVQATDTSIEELGR	−2.33	Organelle and organelle part
42	IPI00830393	Putative uncharacterized protein Cops6	209	7.20E-17	8	31	33855	5.65	SQEGRPMQVIGALIGK;NIEVMNSFELLSHTVEEK;QVCEIIESPLFLK;IGVDHVAR;LILEYVKASEAGEVPFNHEILR;TCNTMNQFVNKFNVLYDR	−2.91	Cellular process and metabolic process
43	IPI00311515	Beta-soluble NSF attachment protein	415	1.80E-37	20	69	33878	5.32	EAVQLMAEAEKR;ASHSFLRGLFGGNTRIEEACEMYTR;NWSAAGNAFCQAAK;HDSATSFVDAGNAYK;HHITIAEIYETELVDIEKAIAHYEQSADYYKGEESNSSANK;VAAYAAQLEQYQKAIEIYEQVGANTMDNPLLK;AALCHFIVDELNAKLALEKYEEMFPAFTDSRLLEAHEEQNSEAYTEAVKEFDSISRLDQWLTTMLLR;	−1.67	Cellular process
44	IPI00322312	Rho GDP-dissociation inhibitor 1	396	1.40E-35	9	42	23450	5.12	SIQEIQELDKDDESLR;YKEALLGRVAVSADPNVPNVIVTR;EGVEYR;YIQHTYR;IDKTDYMVGSYGPR;FTDDDKTDHLSWEWNLTIK	−1000000	Enzyme regulator activity
45	IPI00133853	UPF0687 protein C20orf27 homolog	268	9.00E-23	8	50	19692	6.19	FAAGHDAEGSQSHVHFDEK;YEITFTLPPVR;ETPVHSLHLKLLSVTPTSEGYSIK;EGVLKEEMLLACEGDIGTCVR;HHGTPMLLDGVK	−1.70	Cell part and organelle part
46	IPI00315504	ADP-ribosylation factor-like protein 2	75	0.0016	2	8	21022	5.67	ELQSLLVEER;SHHWR	−1.66	Binding
**Spots 47–56 were up-regulated on both day 14 and day 21**											
47	IPI00129526	Endoplasmin	766	1.40E-72	33	38	92703	4.74	TDDEVVQREEEAIQLDGLNASQIR;FAFQAEVNR;NKEIFLRELISNASDALDKIRLISLTDENALAGNEELTVK;NLLHVTDTGVGMTREELVKEVEEDEYKAFYK;ESDDPMAYIHFTAEGEVTFKSILFVPTSAPRGLFDEYGSKK;RVFITDDFHDMMPKYLNFVKGVVDSDDLPLNVSR;IADEKYNDTFWK;LGVIEDHSNR;FQSSHHSTDITSLDQYVER;QDKIYFMAGSSRKEAESSPFVEREATEKEFEPLLNWMK;LTESPCALVASQYGWSGNMER;DISTNYYASQKKTFEINPR;AYGDRIER	1.54	Metabolic process
48	IPI00123639	Calreticulin	292	3.60E-25	5	16	48136	4.33	EQFLDGDAWTNR;HEQNIDCGGGYVK;IDNSQVESGSLEDDWDFLPPKKIKDPDAAKPEDWDER;GEWKPR	1.66	Immune system process,
49	IPI00131830	Serine protease inhibitor A3K	175	1.80E-13	6	21	47021	5.05	ALYQTEAFTADFQQPTEAK;ISFDPQDTFESEFYLDEKR;HFRDEELSCSVLELK;MQQVEASLQPETLRK;AVLDVAETGTEAAAATGVIGGIR;	1000000	Enzyme regulator activity
50	IPI00131830	Serine protease inhibitor A3K	336	1.40E-29	9	27	47021	5.05	GKTMEEILEGLK;DLQILAEFHEK;ALYQTEAFTADFQQPTEAK;ISFDPQDTFESEFYLDEKR;HFRDEELSCSVLELK;MQQVEASLQPETLRK;AVLDVAETGTEAAAATGVIGGIR	12.57	Enzyme regulator activity
51	IPI00131830	Serine protease inhibitor A3K	316	1.40E-27	8	26	47021	5.05	DLQILAEFHEK;ALYQTEAFTADFQQPTEAK;ISFDPQDTFESEFYLDEKR;HFRDEELSCSVLELK;MQQVEASLQPETLRK;FSIASNYR;AVLDVAETGTEAAAATGVIGGIR	5.29	Enzyme regulator activity
52	IPI00131830	Serine protease inhibitor A3K	187	1.10E-14	5	16	47021	5.05	ISFDPQDTFESEFYLDEKR;HFRDEELSCSVLELK;MQQVEASLQPETLR;AVLDVAETGTEAAAATGVIGGIR	1000000	Enzyme regulator activity
53	IPI00131287	Isoform 1 of N-terminal EF-hand calcium-binding protein 2	374	2.30E-33	11	28	43870	5.18	APLVPDIPSADPGPGPAASRGGTAVILDIFR;KVYEGGSNVDQFVTR;YGGPTPPYIPNHK;TLSFDLQQRLSDEEGTNMHLQLVRQEMAVCPEQLSEFLDSLR;NCFHVAAVR	1000000	Cell part and binding
54	IPI00928020	Putative uncharacterized protein Otub1	331	4.50E-29	7	25	28190	5.15	IQQEIAVQNPLVSER;EYAEDDNIYQQK;YSYIRKTRPDGNCFYR;LLTSGYLQR;FFEHFIEGGR	1.89	Metabolic process
55	IPI00323571	Apolipoprotein E	329	7.20E-29	14	39	35901	5.56	FWDYLR;ELEEQLGPVAEETR;LGADMEDLR;NEVHTMLGQSTEEIR;LSTHLR;LGPLVEQGR;TANLGAGAAQPLR;AQAFGDR;GRLEEVGNQAR;SKMEEQTQQIRLQAEIFQAR;GWFEPIVEDMHR	1000000	Binding and antioxidant activity,
56	IPI00278230	NFU1 iron-sulfur cluster scaffold homolog	131	4.50E-09	5	17	28922	4.92	FIPGKPVLETRTMDFPTPAAAFR;IRPTVQEDGGDVIYRGFEDGIVR	1000000	Organelle and organelle part

**Figure 1:**
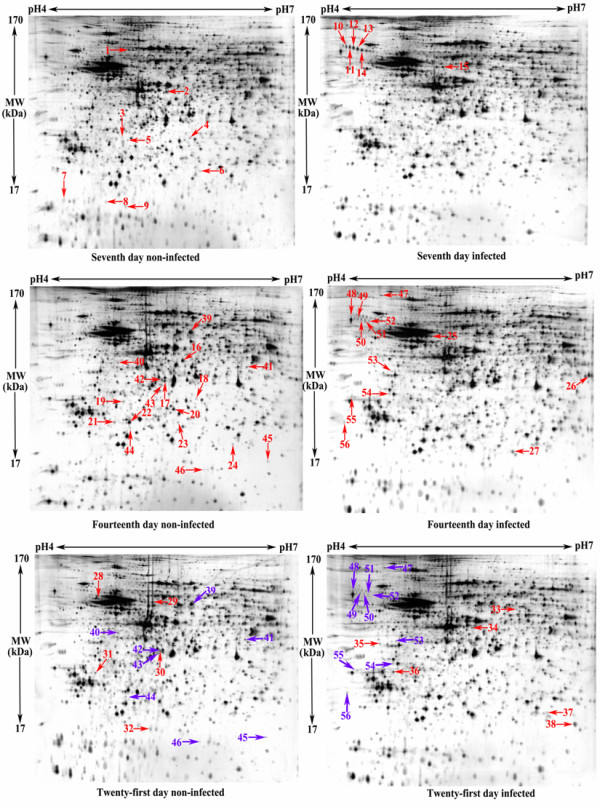
**Two-dimensional electrophoresis profiles of infected and non-infected mouse brain tissues with or without cyst-forming *Toxoplasma gondii*.**

Fifty-six of 60 differentially expressed proteins were successfully identified (Figure 1), corresponding to 45 unique proteins. These proteins are listed in Table 2. Several different protein spots were identified as the same proteins by MS. Spots 4 and 18 were identified as the same protein. Spots 22 and 44 were identified as the same protein. Spots 31 and 36 were identified as different isoforms of the same protein. At 7 d post *T. gondii* infection, 9 spots (spots 1–9) were down-regulated and 6 were up-regulated (spots 10–15) in mouse brain tissue. At 14 d post infection, 17 spots were down-regulated (spots 16–24, 39–46) and 13 were up-regulated (spots 25–27, 47–56). At 21 d post infection, 13 protein spots (spots 28–32, 39–46) were down-regulated and 16 (spots 33–38, 47–56) were up-regulated. Eight protein spots (spots 39–46) and 10 protein spots (spots 47–56) were down- or up-regulated on both 14 d and 21 d, respectively (Figure 1).

### Analysis of differentially expressed proteins

**Figure 2:**
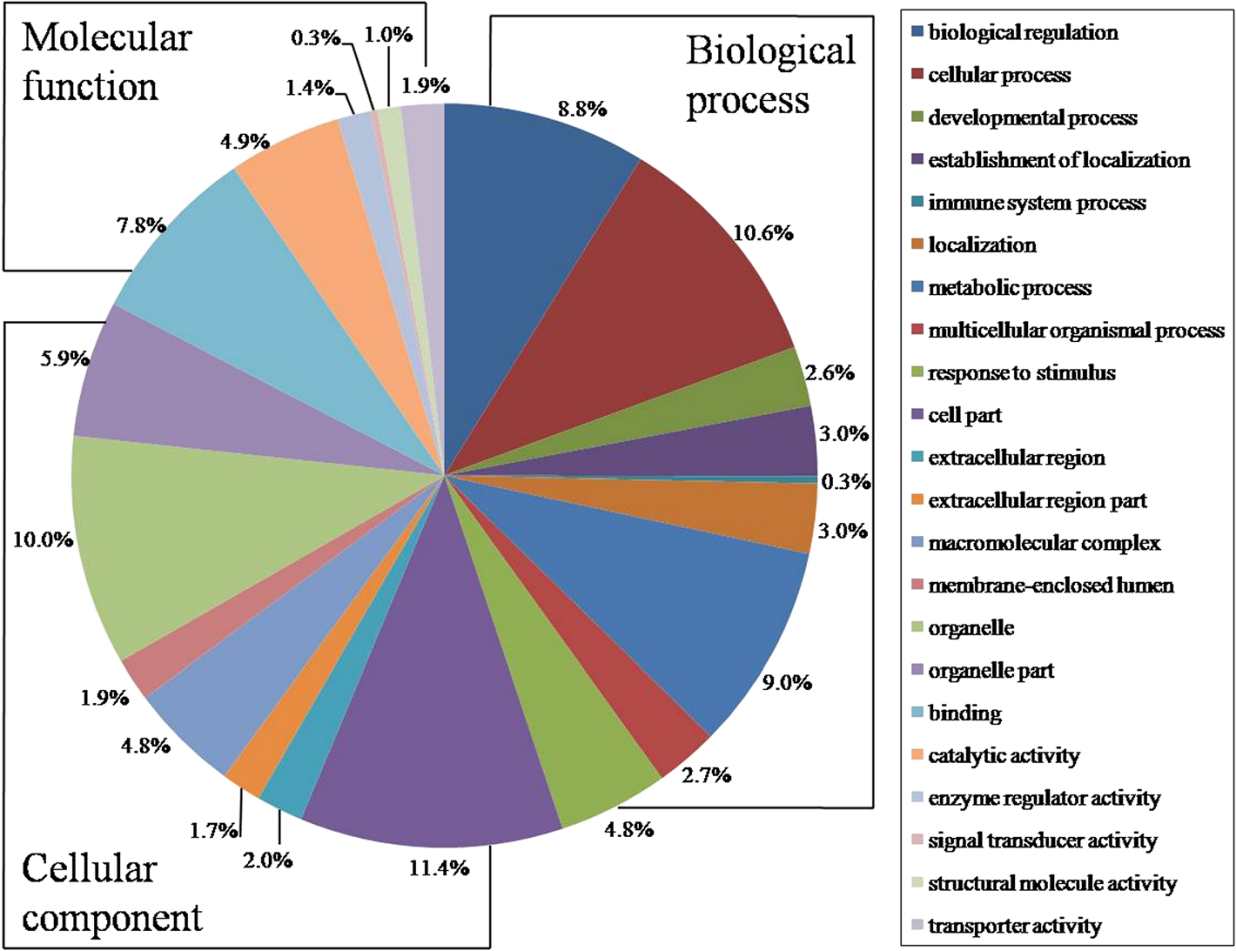
**Gene ontology (GO) categories of the identified proteins were obtained from the Uniprot Web site (**http://www.uniprot.org/**), classified into cellular component, molecular function, and biological process according to the GO terms.**

According to the Uniprot Knowledgebase (Swiss-Prot/TrEMBL) and Gene Ontology database, the functions of successfully identified proteins were grouped based on biological processes, cellular component and molecular function. The results showed that the function of these proteins involved in cellular metabolism, structural molecule activity, immune responses, biological regulation, metabolic process, binding, catalytic activity, enzyme regulator activity, transporter activity and other functions. The differentially expressed proteins were mainly located in the cytoplasm, cell membranes and organelles (mitochondria and lysosomes), and some are secreted proteins (Figure 2).

### Quantitative real-time PCR verification of differentially expressed proteins

Four genes corresponding to the protein spots designated calreticulin, Rho GDP-dissociation inhibitor 1, endoplasmin and stomatin-like protein 2 were chosen for quantitative real-time PCR analysis to quantify their transcript levels. The real time PCR results were consistent with those of the 2-DE studies, and suggested that these proteins identified as differentially expressed were regulated at transcriptional level.

## Discussion

The present study compared the proteomic profiles of brain tissues at 7 d, 14 d and 21 d after infection with cysts of *T. gondii* PRU strain by 2-DE analysis. A total of 60 differentially expressed protein spots were selected and identified by MALDI-TOF MS. Of these, 56 protein spots were successfully identified, which represented 45 different proteins. Four protein spots were not successfully identified, which may be due to the low concentrations of the proteins, which therefore failed to produce high quality mass spectrometric data.

GO analysis revealed that most of the differentially expressed proteins are involved in metabolism, cell structure, signal transduction and immune responses. Here, we focused on the discussion of the functions of several main differentially expressed proteins, and the relationships between these proteins and *T. gondii* infection.

Serine protease inhibitor (SERPIN) A3k is persistently up-regulated in mouse brain tissues 7, 14 and 21 days after infection with *Toxoplasma* cysts. Many members of the serine protease inhibitor superfamily play an important role in the physiological and pathological process, and can be regarded as protease inhibitors which are involved in the coagulation reaction, fiber dissolution, angiogenesis, complement activation, immune and inflammatory reaction [17,18]. It was suggested that SERPINs could inhibit host cell apoptosis [19]. In addition, SERPIN may inhibit replication and decrease *T. gondii* viability [20]. Therefore, the persistence of up-regulated SERPIN A3k may play an important role in preventing the death of infected mouse brain cells as well as limiting the growth of *T. gondii* in the brain tissue cysts.

Protein disulfide isomerase (PDI) is down-regulated 14 days after infection with *T. gondii* cysts. PDI family members can function as molecular chaperones and as disulfide oxidoreductase/isomerases, which means that they can make, break, or rearrange disulfide bonds [21]. The PDI family’s main function is to catalyze the oxidative folding of nascent polypeptide chains in the endoplasmic reticulum, yet they also play an important role in the ER-associated protein degradation pathway (ERAD), protein transport, calcium homeostasis, antigen presentation and virus invasion [22,23]. PDI may play an important role in immune and inflammatory responses and one of the intracellular effector molecules involved in anti-inflammatory reactions [24].

Endoplasmin, also known as heat shock protein 90B1, is a molecular chaperone protein. In the present study, it showed sustained up-regulation in brain tissues 14 and 21 days after infection with *T. gondii*. In Toll like receptor and integrin secretory pathways, it plays a crucial role in folding protein, so was thought to be one of the basic immune chaperone proteins in the regulation of innate and adaptive immunity [25]. Calreticulin (CRT) is persistently up-regulated in brain tissues 14 and 21 days after infection with *Toxoplasma* cysts. Calreticulin is a Ca^2+^ binding protein that has been implicated in many diverse functions inside and outside of the endoplasmic reticulum (ER), including the regulation of intracellular Ca^2+^ homeostasis and Ca^2+^-dependent pathways, chaperone activity, steroid-mediated gene expression, cell adhesion and the interactions of CRT with immunoglobulin G and immunoglobulin Y [26-28]. CRT could be used as a biomarker in lung cancer prediction and diagnosis [29]. CRT also plays an important role in autoimmunity. Enolase 1 and CRT are important proteins in regulating the differentiation and functions of mouse mast cells [30]. CRT has important implications involved in the genesis, development and prognosis of many diseases.

Lamin B1 is down-regulated in mouse brain tissues 7 days after infection with *T. gondii* cysts. Lamins are the important cytoskeletal proteins in the nucleus, and can be divided into two types A and B. Lamin B1 is necessary for growth, development and nuclear membrane integrity in mice [31-33]. Cytochrome b_5_ is also down-regulated in mouse brain tissues 7 days after infection with *T. gondii* cysts. It is a small microsomal protein which serves as an electron transfer component in a number of oxidative reactions in biological tissues, including the anabolic metabolism of fats and steroids, as well as the catabolism of xenobiotics and compounds of endogenous metabolism [34].

Prohibitin (PHB) is down-regulated in mouse brain tissues 14 days after infection with *T. gondii* cysts. PHB has a variety of cell biological functions, including the regulation of cell proliferation, apoptosis, development, transcription, mitochondrial protein folding and as a cell surface receptor [35-37]. In the present study, α-tubulin of mouse brain tissues is up-regulated after infection with *T. gondii* cysts at 21 days post infection, but β-tubulin is down-regulated at 14 days post infection. α- and β-tubulins are the major components of microtubules of the eukaryotic cytoskeleton. Microtubules constitute a major portion of cytoplasmic proteins in nerve cells. They have been implicated to play a central role in axonal transport, neurotransmitter release, neurite outgrowth and synaptogenesis. An increase in the tubulin microheterogeneity was demonstrated during brain maturation. Tubulin comprises a large percentage of the total protein in brain [38-40]. Therefore, abnormal expression of tubulin could have an effect on the normal growth and development of host brain, even causing behavioral disorders of the host.

Apolipoprotein E (ApoE) is persistently up-regulated in brain tissues at 14 and 21 days after infection with *Toxoplasma* cysts. ApoE is one of components of plasma lipoprotein. It regulates the metabolism of plasma lipoprotein by binding to lipoprotein receptors [41]. Abnormal expression of the protein causes the disorder of the metabolism of plasma lipoprotein, and is closely related to atherosclerosis, hypercholesterolemia and hypertriglyceridemia [42].

## Conclusions

The present study revealed changes in the proteomic profiles of mouse brain tissues after infection with cyst-forming *T. gondii* PRU strain (Genotype II). 45 mouse brain proteins differentially expressed between infected and non-infected mice were identified, these proteins were mainly involved in metabolism, cell structure, signal transduction and immune responses. Further exploration of these proteomic data will contribute to understanding the pathogenesis of toxoplasmic encephalitis, and facilitates the discovery of new methods of diagnosis, prevention, control and treatment of *Toxoplasma* encephalopathy.

## Competing interests

The authors declare that they have no competing interests.

## Authors’ contributions

XQZ and DHZ conceived and designed the study, and critically revised the manuscript. DHZ, FRZ and SYH performed the experiments, analyzed the data and drafted the manuscript. MJX, HQS and CS helped in study design, study implementation and manuscript revision. All authors read and approved the final manuscript.
